# Supplementary material for: Revealing druggable cryptic pockets in the Nsp1 of SARS-CoV-2 and other β-coronaviruses by simulations and crystallography
Source: eLife. 2022 Nov 22;11:e81167. doi: 10.7554/eLife.81167 (PMC9681203; doi:10.7554/eLife.81167)
Supplement: Figure 5—source data 1. [file elife-81167-fig5-data1.docx]

| **Selected structures** | **# Consensus clusters** | **# of fragments in the top 3 consensus clusters** | **Pocket location of the top 3 consensus clusters** |
| --- | --- | --- | --- |
| 1 | 7 | 24 | 1 |
|  |  | 17 | 1 |
|  |  | 16 | 3,4 |
| 2 | 8 | 22 | 3,4 |
|  |  | 17 | 1 |
|  |  | 16 | 1 |
| 3 | 12 | 17 | 1 |
|  |  | 17 | 3 |
|  |  | 14 | 1 |
| 4 | 7 | 22 | 1 |
|  |  | 18 | 3,4 |
|  |  | 16 | 1 |
| 5 | 11 | 20 | 1 |
|  |  | 14 | 3,4 |
|  |  | 13 | 1 |
| 6 | 11 | 16 | 1 |
|  |  | 14 | 3 |
|  |  | 11 | 2 |
| 7 | 10 | 16 | 1 |
|  |  | 16 | 1 |
|  |  | 15 | 4 |
| 8 | 11 | 16 | 1 |
|  |  | 15 | 3,4 |
|  |  | 13 | 1 |
| 9 | 8 | 15 | 1 |
|  |  | 19 | 1 |
|  |  | 16 | 3,4 |
| 10 | 11 | 18 | 1 |
|  |  | 16 | 1 |
|  |  | 15 | 1 |
| 11 | 11 | 25 | 1 |
|  |  | 16 | 1 |
|  |  | 15 | 2 |

**Figure 5 – Source data 1.** Consensus clusters obtained from the FTMap program.
