# Supplementary material for: Revealing druggable cryptic pockets in the Nsp1 of SARS-CoV-2 and other β-coronaviruses by simulations and crystallography
Source: eLife. 2022 Nov 22;11:e81167. doi: 10.7554/eLife.81167 (PMC9681203; doi:10.7554/eLife.81167)
Supplement: Figure 6—source data 1. [file elife-81167-fig6-data1.docx]

|  | **Nsp1-2E10** |
| --- | --- |
| **PDB ID** | 8A4Y |
| **Wavelength [Å]** | 0.96546 |
| **Resolution range [Å]** | 35.50 - 1.10 (1.14 - 1.10) |
| **Space group** | P4_3_2_1_2 |
| **Unit cell parameters (Å, °)** | *a*=*b*=36.688, *c*=140.928, α=β=γ=90 |
| **Total reflections** | 464757 (27903) |
| **Unique reflections** | 40442 (3926) |
| **Multiplicity** | 11.5 (7.1) |
| **Completeness [%]** | 99.9 (98.9) |
| **Mean I/sigma(I)** | 18.4 (2.6) |
| **Wilson B-factor (Å^2^)** | 12.96 |
| **R-meas [%]** | 6.7 (65.9) |
| **R-pim [%]** | 1.9 (24.1) |
| **CC_1/2_ [%]** | 99.8 (85.4) |
| **R_cryst_/R_free_ [%]** | 14.8 (19.6) / 15.8 (18.9) |
| **Total no. of non-hydrogen atoms (protein)** | 1082 |
| **No. of protein/ligand/solvent atoms** | 931 / 26 / 138 |
| **Average B-factor/protein/ligands/solvent** | 22.0 / 19.7 / 46.9 / 34.7 |
| **RMSD (bonds, angles)** | 0.012 / 1.57 |
| **Ramachandran**  **favored/allowed/outliers/rotamer outliers [%]** | 97.4 / 2.6 / 0/0 / 2.9 |
| **Clashscore** | 4.2 |

**Figure 6 – Source data 1.** Data collection, data processing, and model refinement statistics for the SARS CoV-2 Nsp1_N_-2E10 complex. Data in parenthesis correspond to the highest resolution shell.
