# Supplementary material for: Revealing druggable cryptic pockets in the Nsp1 of SARS-CoV-2 and other β-coronaviruses by simulations and crystallography
Source: eLife. 2022 Nov 22;11:e81167. doi: 10.7554/eLife.81167 (PMC9681203; doi:10.7554/eLife.81167)
Supplement: Peer review history

# Editor report: Editor's evaluation

**DOI:** 10.7554/eLife.81167.sa0

**Authors:** Yogesh K Gupta

**Affiliations:** https://ror.org/02f6dcw23 The University of Texas Health Science Center at San Antonio United States

SARS-CoV-2 nonstructural protein (Nsp1) has emerged as an attractive target as it plays an important role in modulating the host and viral gene expression. This study describes multiple druggable sites in Nsp1. A 1.1Å co-crystal structure of Nsp1 with a fragment, together with computational studies, provides a framework for the rational design of potential antiviral candidates. This important study is methodologically convincing and will be of interest to researchers in the fields of structural virology and rational drug design.

# Decision letter

**DOI:** 10.7554/eLife.81167.sa1

**Affiliations:** https://ror.org/02f6dcw23 The University of Texas Health Science Center at San Antonio United States; https://ror.org/049s0rh22 Dartmouth College United States

Our editorial process produces two outputs: (i) public reviews designed to be posted alongside the preprint for the benefit of readers; (ii) feedback on the manuscript for the authors, including requests for revisions, shown below. We also include an acceptance summary that explains what the editors found interesting or important about the work.

**Decision letter after peer review:**

Thank you for submitting your article "Revealing druggable cryptic pockets in the Nsp-1 of SARS-CoV-2 and other β-coronaviruses by simulations and crystallography" for consideration by *eLife*. Your article has been reviewed by three peer reviewers, one of whom is a member of our Board of Reviewing Editors, and the evaluation has been overseen by Volker Dötsch as the Senior Editor. The following individual involved in the review of your submission has agreed to reveal their identity: Paul Robustelli (Reviewer #3).

The reviewers have discussed their reviews with one another, and the Reviewing Editor has drafted this to help you prepare a revised submission.

Essential revisions:

The reviewers agree that your study demonstrates state-of-the-art computational methods for discovering experimentally verifiable drug-binding pockets and fragment hits. However, they also raised some questions. Please address the concerns raised by the three reviewers and submit a revised version. The comments are appended to this email.

*Reviewer #1 (Recommendations for the authors):*

1) The binding affinity of Nsp1/E210 interaction was measured by MST and TSA, but the data were missing. Can authors include these data in Figure 6 (or as a supplement figure)?

2) Nsp1 blocks the mRNA channel in the ribosome to shut off protein translation. The virus must utilize the host translation machinery to synthesize its proteins. How is the Nsp1-mediated translation block lifted during the synthesis of viral proteins? How does Nsp1 binding to the virus's SL1 mRNA benefit the virus? The virus likely uses different capabilities of Nsp1 at different stages of infection to maximize its survival. The authors should consider expanding the discussion by clarifying some of these points.

*Reviewer #2 (Recommendations for the authors):*

I have the following concerns that need to be addressed:

1) The cryptic pocket was identified using the atomic coordinates from the crystal structure of Nsp1-N (10-126 aa), where the C-terminal residues are missing. Figure 3 shows that Pocket 1 is close to the C-terminal end of Nsp1-N (R124, K125, N126). This raises the question about this cryptic pocket in the context of full-length Nsp1. Whether the C-terminal tail would mask this pocket or not? And whether it will be accessible to the drugs in the full-length Nsp1.

2) The binding of the drug to full-length Nsp1 is weak (Kd value of 2.74 {plus minus} 2.63 mM). Further, the associated error is almost the same as the Kd value. Computational drug/fragment binding studies were done with Nsp1-N. Biophysical interaction studies were done with full-length Nsp1, which showed weak binding. Is it possible that the C-terminal is masking the pocket and hence weaker binding? It would be interesting to check the drug binding with the C-terminal truncated Nsp1.

3) The authors mention that targeting the identified pocket in Nsp1-N could disrupt the SARS-CoV-2 Nsp1-mRNA complex. However, there are conflicting reports on direct binding between Nsp1-mRNA (See references 17 and 29).

4) It would be interesting to compare the drug-bound Nsp1-N with that of mRNA-bound Nsp1-N to check if the conformational changes explain how the binding of the drug could potentially disrupt the Nsp1-mRNA complex.

5) Figures 2 and 3 show that Nsp1-N is slightly different orientations. It will be helpful for the reader if the figures show similar orientations. (Also, for Figure 4, if all 4 pockets can be shown clearly in the above orientations).

6) Show electron density for the drug bound to Nsp1 in the PanDDA map and unbiased experimental map.

*Reviewer #3 (Recommendations for the authors):*

1) This manuscript would be strengthened by the inclusion of additional data regarding the 60 potential fragment hits tested by crystal soaking. It would be nice to see the structures/SMILES strings of the 60 fragments that were tested, along with information about their predicted binding sites, or perhaps even a PDB of each predicted binding mode. I feel that while Figure 5 is informative, it would be helpful to be able to better understand the nature of the proposed binding modes to pockets 2-4, and more cryptic regions of pocket 1 in more detail.

2) It would also be valuable to include the 25 representative MD conformations that were used to search for fragment hits.

3) While the authors have identified crystal contacts that may have prevented fragment binding in crystal soaking experiments, in terms of validating the claims of the identification of druggable cryptic binding pockets, it would be quite interesting if the authors could test a subset of the other fragments with orthogonal biophysical assays (such as MST, TSA or other assays) to determine if there is evidence that fragments that bind to pockets 2-4 and more cryptic regions of pocket 1 bind.

# Reply: Author response

**DOI:** 10.7554/eLife.81167.sa2

Reviewer #1 (Recommendations for the authors):

1) The binding affinity of Nsp1/E210 interaction was measured by MST and TSA, but the data were missing. Can authors include these data in Figure 6 (or as a supplement figure)?

The data has been included in the revised manuscript: Table 2 and Figure 6 —figure supplement 2 show the TSA and the MST results, respectively.

2) Nsp1 blocks the mRNA channel in the ribosome to shut off protein translation. The virus must utilize the host translation machinery to synthesize its proteins. How is the Nsp1-mediated translation block lifted during the synthesis of viral proteins? How does Nsp1 binding to the virus's SL1 mRNA benefit the virus? The virus likely uses different capabilities of Nsp1 at different stages of infection to maximize its survival. The authors should consider expanding the discussion by clarifying some of these points.

To address this point, the following lines were added to the introduction and conclusion sections:

Introduction:

“The proposed mechanism suggests that Nsp1 plays the role of a ribosome gatekeeper by sterically blocking the mRNA tunnel of the ribosome for the host mRNAs and the blockage is lifted upon the interaction of Nsp1 with the 5’-UTR SL1 sequence of the viral mRNA. In this way, the virus can inhibit selectively the translation of the host mRNA and highjack the ribosome to foster the translation of its own mRNA.^18,19”^

Conclusion:

“Moreover, we show how the fragment bound to one of these pockets can disrupt the Nsp1-mRNA complex. In particular, the binding of the fragment to the identified pocket interferes with crucial interactions between the 5’-UTR SL1 and Nsp1, namely R124 and K125. This could prevent the viral mRNA to be efficiently translated, ultimately impairing the viral translation strategy. At the same time, the fragment is expected also to lower the affinity of Nsp1 for the ribosome by hindering the interaction mediated by R99, R124 and R125.”

Reviewer #2 (Recommendations for the authors):

I have the following concerns that need to be addressed:

1) The cryptic pocket was identified using the atomic coordinates from the crystal structure of Nsp1-N (10-126 aa), where the C-terminal residues are missing. Figure 3 shows that Pocket 1 is close to the C-terminal end of Nsp1-N (R124, K125, N126). This raises the question about this cryptic pocket in the context of full-length Nsp1. Whether the C-terminal tail would mask this pocket or not? And whether it will be accessible to the drugs in the full-length Nsp1.

To address the reviewer’s comment, we ran additional unbiased MD simulations starting from the full-length Nsp1 in water (see Methods section in the revised manuscript). Moreover, we repeated the MST experiments on the full-length protein. Both the simulations and the experiments indicate that pocket 1 is still accessible in the full-length protein. Based on the obtained results, the following paragraph was added to the revised manuscript (lines 332-352),

To assess whether the C-terminal end would affect the accessibility of a fragment to pocket 1, we quantified the volume of the pocket throughout three independent replicas. The C-terminal domain indeed affects the accessibility of the identified pocket. The distribution of the pocket volume (see revised manuscript Figure 8 —figure supplement 3) shows how the volume of pocket 1 reduces to approximately 200 Å^3^ in the case of Nsp1_FL_, reaching similar values to the ones found in the crystal structure of the apo Nsp1 (PDB entry 7K7P). The loop that connects the N- to the C-terminal (aa Arg124-Pro153) is flexible and a portion of it (aa Arg124-Gly137) occasionally occludes pocket 1. Nonetheless, we clustered the last 200 ns of the trajectories and extracted representative structures per replica corresponding to the most populated clusters. For the structure representing the second most populated cluster in replica 2, we were able to dock the fragment within pocket 1, obtaining a similar pose to the one seen in the presented crystal structure (Figure 8 —figure supplement 3). This indicates that, even in the setting of full-length Nsp1, pocket 1 is still accessible in the context of the full-length Nsp1. Moreover, we obtained a docked pose also for replica 3, even if the site is partially closed due to the inward orientation of Arg43 (Figure 8 —figure supplement 3). A conformation where the pocket was fully closed was instead obtained for replica 1, where no fragment could be docked to the pocket. Taken together, these results indicate that pocket 1 is still accessible for fragment binding in our full-length Nsp1 model in solution. It is worth noting that the flexible nature of the loop surrounding the pocket supports the hypothesis of the cryptic nature of the site, which is partially detectable in the crystal structures where the C-terminal domain is absent. Ultimately, the accessibility predictions from our simulations are confirmed by the MST measurements conducted on Nsp1_FL_ (Figure 6 —figure supplement 2). The measured KD has a larger error bar, but within the error is comparable to the one obtained for Nsp1_N_, indicating that the C-terminal domain present in Nsp1_FL_ does not prevent the binding of the fragment.

On a model of the full-length Nsp1 that we built based on the cryo-EM structure (PDB entry 7JQB) of the Nsp1 C-terminal bound to the ribosome, we see how the C-terminal tail is buried inside the ribosome (Author response image 1A), extending away from the N-terminal (Author response image 1B). In this model, the N-terminal end is free to adopt at least two different orientations and occupy the accessible space on the surface of the ribosome (Author response image 1C, R.D). Out of the two conformations shown in Author response image 1C, we think that the one in which the N-terminal is closer to the ribosomal protein S3 might be more favourable as it minimises the clashes with the RNA, while making residue R99 more accessible for interaction with the ribosome (e.g. E74 and Y57 of the ribosomal protein S3). What is more, superposition of our crystal structure of the identified fragment bound to the N-terminal end of Nsp1 to the either of these conformations of this model shows that the binding site is exposed to the solvent and accessible for binding in both conformations (Author response image 1E). We acknowledge that this is a model based solely on the available structural data to this day and more extensive studies are needed to reveal the dynamics of the Nterminal with respect to the ribosome and RNA. Nevertheless, this model already indicates that the C-terminal tail is not expected to interfere with the binding of a molecule to pocket 1 in the Nsp1-ribosome context.

**Author response image 1.** Model of the full-length Nsp1 based on the cryo-EM structure of the C-terminal bound to the ribosome (PDB entry 7jqb). .

2) The binding of the drug to full-length Nsp1 is weak (Kd value of 2.74 {plus minus} 2.63 mM). Further, the associated error is almost the same as the Kd value. Computational drug/fragment binding studies were done with Nsp1-N. Biophysical interaction studies were done with full-length Nsp1, which showed weak binding. Is it possible that the C-terminal is masking the pocket and hence weaker binding? It would be interesting to check the drug binding with the C-terminal truncated Nsp1.

We also refer the reviewer to point (1). Generally, initial fragment hits show KD values in the low mM range, consistent with our experimental data. The high error bars are due to the weak binding. Moreover, ligand-induced fluorescence can affect the results. We further optimised the experimental conditions and remeasured the binding affinity both in Nsp1_N_ and in the full-length protein. The new measurements bring down the error bar for Nsp1_N_ and show that the binding affinity of the fragment is comparable to that in the full-length Nsp1, within the error. However, after correcting for the ligand-induced fluorescence, we find a value that is somewhat weaker than the original one. The new MST data is shown on page 24 in Figure 6 —figure supplement 2.

3) The authors mention that targeting the identified pocket in Nsp1-N could disrupt the SARS-CoV-2 Nsp1-mRNA complex. However, there are conflicting reports on direct binding between Nsp1-mRNA (See references 17 and 29).

It is indeed true that the mechanism of action of Nsp1 and the exact sequence of events that allow the translation of the viral mRNA with the help of Nsp1 are still under debate. While Vankadari et al. claim that the SL1 of the 5’-UTR region of the viral mRNA is recognised by Nsp1, which then recruits the host ribosome at that site (ref. 17), increasing evidence suggests that free Nsp1 has no affinity for mRNA, even when the mRNA contains SL1 like the SARS-CoV-2 5′UTR, and mRNA binding and cleavage occur only in the Nsp1-ribosome complex (ref. 18, 19).

In a recent study, Afsar et al. (ref. 21) showed that inhibition of Nsp1 through a compound that binds to its C-terminal domain can reduce the expression of the viral spike protein in HEK293T-ACE2 and Vero-E6 cells. This result implies that regardless of whether Nsp1 is inhibited before binding to the ribosome or whilst in the complex with it, targeting Nsp1 is a viable antiviral approach.

Overall, available evidence suggests that targeting the N-terminal domain of Nsp1 is a viable approach because regardless of whether Nsp1 binds to the SL1 first and then to the ribosome or the other way round, in both cases the mRNA will still have to bind to the Nsp1 for its translation to take place. This means that the binding site of mRNA should be accessible and not blocked by the ribosome. To the best of our knowledge, to this day there is no structure of the N-terminal region Nsp1 bound to the ribosome. However, mutagenesis data regarding the important interactions between Nsp1, mRNA, and the ribosome suggest that residues R99, R124, K125 are involved in the interaction with the ribosome while at the same time being crucial for the interaction with the mRNA (ref. 19). Since according to our Nsp1_FL_-RNA model (Figure 8), our fragment interacts with residues that have been described as crucial for the interaction of Nsp1 with mRNA and the ribosome (ref. 19), we think that molecules that would bind to the same site as the fragment would exert their action by competing with the mRNA for binding to Nsp1, regardless of whether this competition happens whilst the Nsp1 is still free or in complex with the ribosome.

4) It would be interesting to compare the drug-bound Nsp1-N with that of mRNA-bound Nsp1-N to check if the conformational changes explain how the binding of the drug could potentially disrupt the Nsp1-mRNA complex.

We thank the reviewer for the comment, and we refer them to lines 315-331 (pages 9-10) of the manuscript. There, we discuss how the biding of the fragment to pocket 1 of the Nsp1_N_ changes the orientation of the sidechain of certain residues and how this could affect the Nsp1_FL_-mRNA complex. The change in these residues is evident after superimposing the drug-bound Nsp1_N_ with that of the mRNA-bound Nsp1_FL_ complex as suggested by the reviewer. We considered the full-length Nsp1 in our Nsp1-mRNA model as some of the residues (aa 122-130) of the C-terminus of Nsp1 were shown to have a function role, probably by establishing contacts with the viral mRNA (ref. 19).

5) Figures 2 and 3 show that Nsp1-N is slightly different orientations. It will be helpful for the reader if the figures show similar orientations. (Also, for Figure 4, if all 4 pockets can be shown clearly in the above orientations).

We thank the reviewer for the suggestion. We changed in the revised manuscript the protein’s orientation in Figure 3 and Figure 4 to be the same as in Figure 2.

6) Show electron density for the drug bound to Nsp1 in the PanDDA map and unbiased experimental map.

The data has been added in Figure 6—figure supplement 1 of the revised manuscript.

Reviewer #3 (Recommendations for the authors):

Regarding the reviewer’s concern about the cryptic nature of the identified pocket, we refer the reviewer to our response to Reviewer #2, point 1, and lines 332-352 of the revised manuscript, where we addressed this with additional MD simulations and MST experiments on the full-length Nsp1.

1) This manuscript would be strengthened by the inclusion of additional data regarding the 60 potential fragment hits tested by crystal soaking. It would be nice to see the structures/SMILES strings of the 60 fragments that were tested, along with information about their predicted binding sites, or perhaps even a PDB of each predicted binding mode. I feel that while Figure 5 is informative, it would be helpful to be able to better understand the nature of the proposed binding modes to pockets 2-4, and more cryptic regions of pocket 1 in more detail.

In the revised manuscript we have included a link (page 29, line 926) to a GitHub repository containing the proposed binding modes. Moreover, we also added a table (Table 1) with the SMILES and the predicted binding sites for each tested fragment. No data was included for pocket 2 since this pocket was not considered for the fragment screening. We erroneously reported that we tested 60 fragments, we actually tested 59. The number was corrected in the revised manuscript.

2) It would also be valuable to include the 25 representative MD conformations that were used to search for fragment hits.

We have included a link in the revised manuscript (page 15, line 518) to a GitHub repository through which the reader can download these conformations.

3) While the authors have identified crystal contacts that may have prevented fragment binding in crystal soaking experiments, in terms of validating the claims of the identification of druggable cryptic binding pockets, it would be quite interesting if the authors could test a subset of the other fragments with orthogonal biophysical assays (such as MST, TSA or other assays) to determine if there is evidence that fragments that bind to pockets 2-4 and more cryptic regions of pocket 1 bind.

This is a very good point; indeed, we have tested the 59 potential fragments hits emerging from computational screening and the data summarised in the new Table 2 in the revised manuscript. As can be seen, most fragment hits show destabilisation of Nsp1 or have no effects on the protein’s stability. We conclude from these experiments that TSA is not yet useful as an orthogonal biophysical assay to confirm binding and stabilisation by fragments. This is in agreement with many reports from academia and industry on other fragment-based screening studies, as well with our own previous conclusions on another SARS-CoV-2 protein, non-structural protein 10 (Nsp10) for which we obtained similar results. Therefore, a shift for the crystallographic hit (reported here) and for other hits binding to pocket 1 verified by crystallography and reported in ref. 22 are not measurable. Thus, we assume that thermal shift assays will become useful for measuring stabilisation, once molecules are larger and display higher affinity. Albeit one of the fragments predicted to bind to pocket 4 shows a stabilizing effect, given the lack of shift for the crystallographic hits, we cannot confidently conclude that the predicted binding to pocket 4 is indeed real based solely on TSA. With respect to MST, the experiments are very time consuming and expensive and so we generally only perform these on confirmed structural hits. We thus did an MST assay on the fragment predicted to bind to pocket 4 that showed a stabilising effect but the measured KD value was > 15 mM. As authors we appreciate that the potential fragment hits that are predicted to bind to pockets 2-4 by docking would need to be further investigated and developed into leads to confirm their affinity and drug design potential. Still, here we prioritised the hits we succeeded in obtaining structural data for.
